# Posterior Alien Hand Syndrome from Acute Ischemic Left Parietal Lobe Infarction

**DOI:** 10.7759/cureus.5828

**Published:** 2019-10-03

**Authors:** Gaurav Gheewala, Rajan Gadhia, Salim R Surani, Iqbal Ratnani

**Affiliations:** 1 Anesthesiology and Critical Care, Houston Methodist Hospital, Houston, USA; 2 Neurology, Houston Methodist Neurological Institute, Houston, USA; 3 Internal Medicine, Texas A&M Health Science Center, Temple, USA

**Keywords:** alien hand syndrome, involuntary movement, parietal lobe, stroke, middle cerebral artery

## Abstract

Alien hand syndrome (AHS) is defined as an involuntary goal-directed movement of the hand as if acting on its own will, or as being under the control of someone else. Moreover, the affected hand typically does not show any signs of weakness or convulsive movement. The cause of AHS is associated with an insult to the brain from various conditions such as stroke, trauma, tumor, aneurysm, neurosurgical intervention, infection, and degenerative brain diseases. We hereby illustrate a case of a patient with chronic atrial dysrhythmia whose oral anticoagulation therapy was placed on hold by his gastroenterologist for a scheduled colonoscopy. The patient presented to the hospital with symptoms of right-hand paresthesia with uncontrolled movement. These symptoms were seen along ST-segment elevation in the inferior leads on a 12 lead electrocardiogram. The case report acknowledges an unusual presentation of acute ischemic stroke, which may be frightening and bewildering to patients, their families, and any healthcare providers, including neurologists, who may have encountered it for the first time. Also, our patient had posterior AHS, likely from infarction involving the left inferior parietal lobe, which is reported to have a low prevalence.

## Introduction

Alien hand syndrome is a rare neurological disorder, which typically affects the left hand. The first case of alien hand syndrome (AHS) was published by Kurt Goldstein in 1908 as the “doctrine of motor apraxia.” AHS patients are aware that the hand belongs to them, but some external forces seem to have control, which may be a source of annoyance for them. Even though it is truly an objective neurological symptom, it is often misdiagnosed as a psychiatric disorder due to its clinical presentation, which often delays appropriate time-sensitive treatment. Based on the involved part of the brain and its symptoms, AHS can be classified into several different categories: alien hand sign, sensory alien hand syndrome, anarchic hand, diagnostic dyspraxia, levitating hand, supernumerary hands, agnostic dyspraxia, and many more [1].

## Case presentation

**Figure 1:**
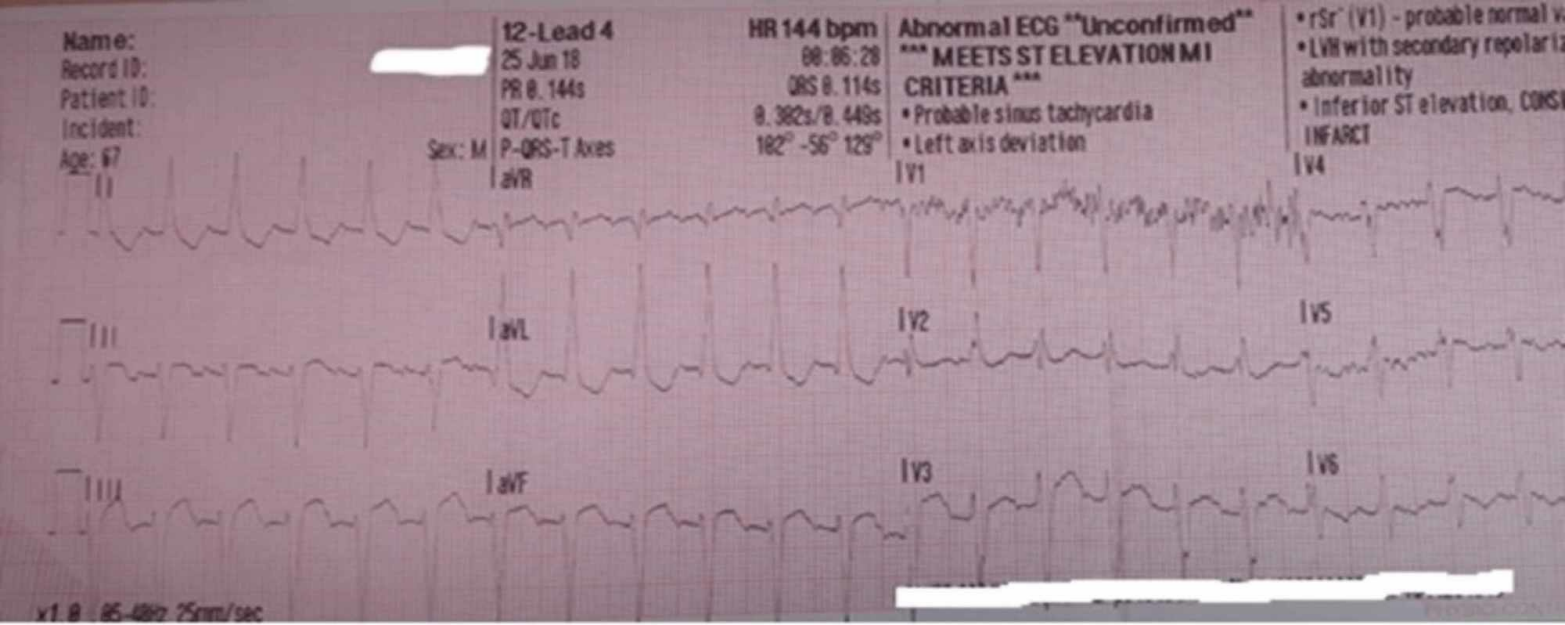
Initial ECG performed by EMS Inferior leads ST elevation with HR 144 bpm

This case report describes a 67-year-old right-handed male patient with a past medical history of hypertension, hyperlipidemia, diabetes mellitus type 2, diastolic heart failure, chronic atrial fibrillation/atrial flutter, chronic right posterior cerebral artery (PCA) stroke with residual left visual field deficits, and seizures. Upon request of his gastroenterologist he stopped taking his oral anticoagulation therapy (OAT), apixaban, for a scheduled colonoscopy due to rectal bleeding. On the fifth day of stopping apixaban, he was brought to the emergency department (ED) by emergency medical services (EMS) with vague complaints of difficulty with bilateral hand coordination, right-hand paresthesia with intermittent involuntary touching of his face, with normal grip strength. He was also found to have gait impairment. The patient denied any chest pain, pressure or heaviness, but an electrocardiogram (ECG) which was done by EMS, showed ST-segment elevation in inferior leads. His heart rhythm was in supraventricular tachycardia with heart rate (HR) in 140 bpm range, and therefore, code ST-elevation myocardial infarction (STEMI) was activated (Figure 1).

**Figure 2:**
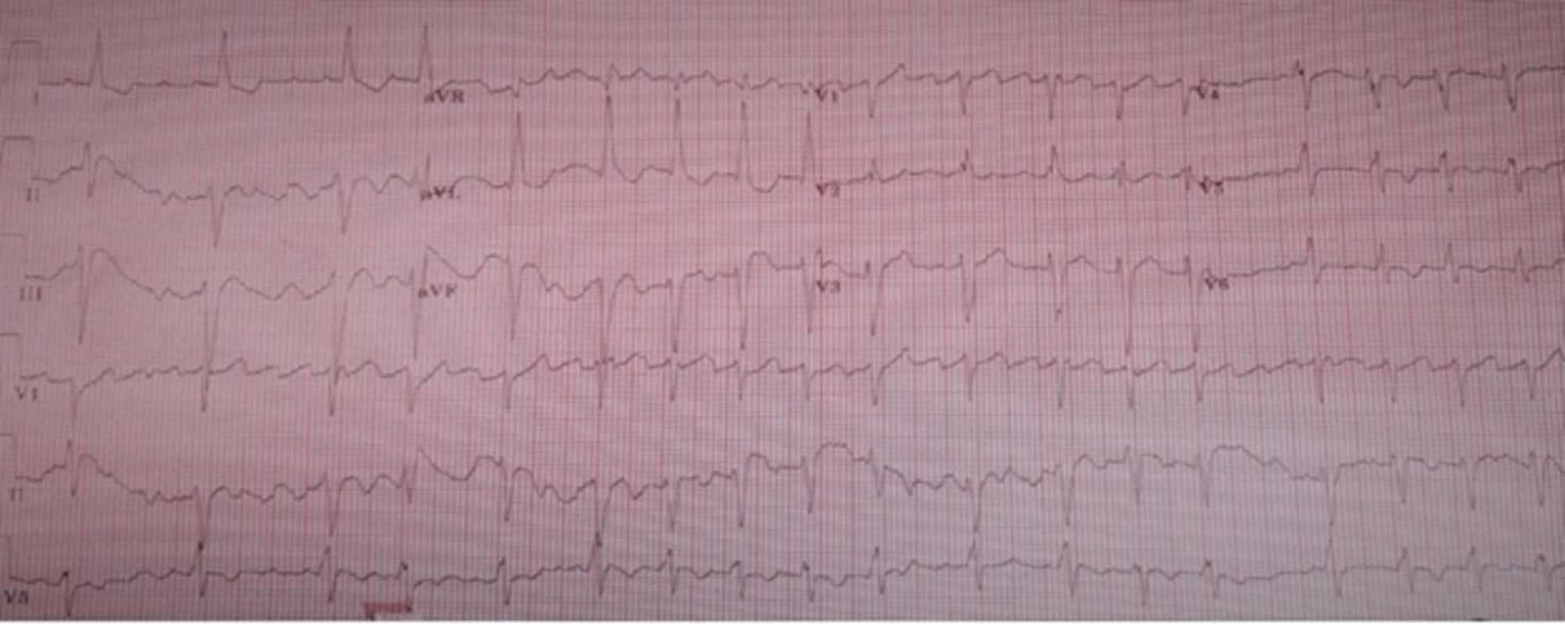
ECG upon arrival to hospital Atrial flutter with premature ventricular contractions with HR 112

While in the ED, repeat ECG showed similar findings, but the heart rhythm indicated atrial flutter with HR 112 bpm (Figure 2).

**Figure 3:**
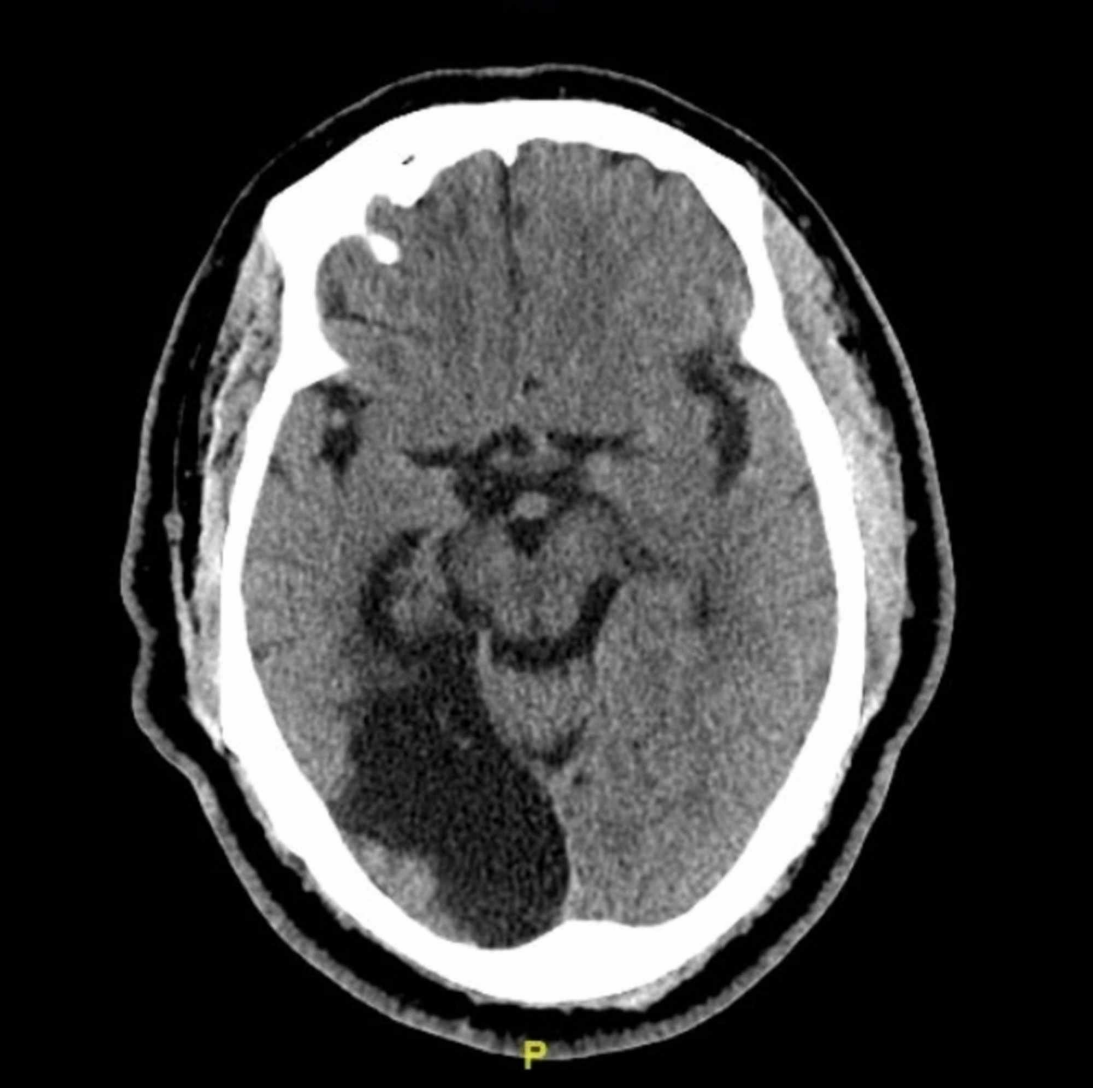
CT scan of brain without contrast Old right posterior cerebral artery territorial infarct involving the occipital and temporal lobes

Due to the stroke-like symptoms, the patient first underwent computed tomography (CT) scan of the head without contrast which was unremarkable for acute findings, and notable only for a chronic right PCA infarct involving the occipital and temporal lobes (Figure 3).

**Figure 4:**
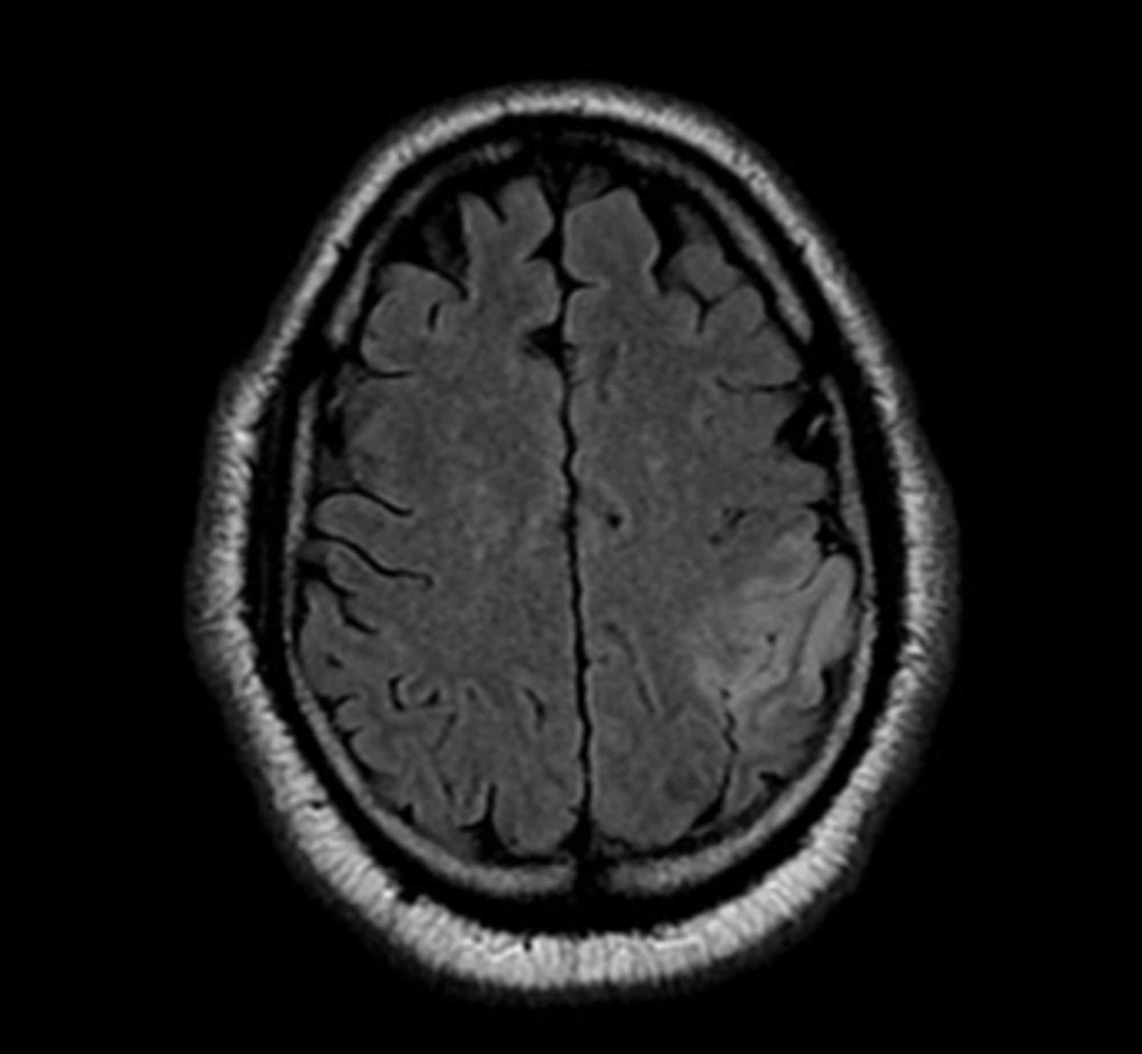
MRI of the brain without contrast: Axial T2 flair There is an area of restricted diffusion seen in the left inferior parietal lobe, including the postcentral gyrus, as well as associated increased signal intensity on FLAIR consistent with acute ischemic insult in left middle cerebral artery (MCA) vascular territory.

Given that no acute abnormalities were identified on CT scan, the patient underwent an emergent coronary angiogram, which showed preserved left ventricular function with no significant coronary artery disease. Cardiac enzymes returned normal and transthoracic echocardiogram of the heart confirmed normal biventricular functions and did not show any evidence of thrombus. Post-procedure, the patient was admitted to the intensive care unit and the neurology team was consulted as he continued to have the above-mentioned neurological deficits. In addition, he was found to have impairment with repetition and inconsistent errors in articulation with dysfluency of speech. The remainder of his sensory and motor neurological exam was unremarkable. The neurological consult was not convinced that the patient was having typical signs or symptoms of a stroke or seizure, given that his symptoms were inconsistent and intermittent. Since the patient and his wife kept insisting that such behavior of the patient was not his neurological baseline, magnetic resonance imaging (MRI) of the brain without contrast was performed. This showed a new acute left inferior parietal lobe infarction, including postcentral gyrus, likely from left middle cerebral artery territory ischemia (Figure 4).

Magnetic resonance angiogram (MRA) of the head and neck did not show significant stenosis or aneurysm of major vessels. Given that the patient was out of the typical intravenous thrombolytic window from when he was last known to be well, he did not receive IV tPA (tissue plasminogen activator). Later, an electroencephalogram (EEG) was performed to rule out epileptiform activity, which showed unremarkable results. Over the next day, the patient recovered from the right-hand involuntary movement. He was diagnosed as having an acute ischemic stroke likely from atrial dysrhythmia and ST elevation myocardial infarction (STEMI) was ruled out. Additionally, when his ECG was compared to the previous admission ECG, it did not show any significant new changes. That leads us to believe that A-flutter waveforms mimicked inferior wall myocardial infarction. For stroke, aspirin was started on the same day of admission; in addition, OAT was restarted on the fourth day of admission.

## Discussion

Alien hand syndrome is also called foreign hand syndrome, or Dr. Strangelove syndrome, as the affected hand behaves in the manner of a third person or as if the foreign object is controlling it. There are two criteria that define AHS: first, the hand should move involuntarily and second, the hand should act as if it has its own will [2]. As mentioned previously, the causes of AHS are generally associated with an insult to various locations in the brain and from various conditions nevertheless, AHS from stroke is rare. Many studies are available which describe AHS resulting from anterior cerebral artery or posterior cerebral artery infarction as they deliver blood to the corpus callosum [1]. Damage to corpus callosum leads to interhemispheric disconnection, therefore, disrupting the ability to inhibit the activity of the contralateral hemisphere. This is the perceived mechanism for involuntary motor activity of the contralateral side [3]. 

Typically, AHS is associated with an insult to the corpus callosum or frontal lobe, but in a paucity of cases, it is related to parietal or occipital lobes involvement. When AHS is associated with parietal lobe, occipital lobe, or thalamus, it is called posterior AHS. Any cause for disturbance to the normal function of parietal and occipital lobes not only produces less complex motor activities, but also causes some sensory impairment compared to the other forms of AHS [3]. The precise pathophysiology is unknown regarding how parietal lobe disruption can lead to AHS, although it is believed to be from error in neuronal connections. The inferior parietal lobe receives input from various neuronal pathways and coordinates motor output, thus, impairment of this area can lead to inappropriate release of motor activity without necessary check from the sensory stimuli [1, 4]. In our case, this patient had involuntary movement of right hand and arm, paresthesias, as well as dysphasia and difficulty with repetition, which indicated to us that he had both motor and sensory involvement. This is more typical of what is seen in posterior AHS. In addition, neuroimaging studies endorsed infarction of the left parietal region, confirming that this patient’s AHS was likely related to this area of acute ischemia. Furthermore, posterior AHS is also called sensory AHS, due to the involvement of both sensory and motor pathways.

The diagnosis of AHS remains complicated as it may have some overlap with symptoms seen in patients with behavioral disorders. In 2007 functional MRI (fMRI) was developed by Swiss doctors to assist in studying part of the brain involved during voluntary versus involuntary movement in AHS patients. Voluntary movements were found to have complex neuronal pathways with the contralateral primary motor cortex, connections between bilateral frontal, and parietal cortices. On the contrary, the involuntary movement was only activated within the primary motor cortex, leading to a subconscious goal-directed movement of hand [5]. Since the exact pathophysiology of AHS is unknown, it has been difficult to treat AHS. In AHS patients, benzodiazepines and botulinum toxin injections are useful in relieving anxiety and fear. But, the most essential part of management is to identify functional limitations from AHS and promote the use of behavioral, physical, and occupation therapies to adapt to the new limitations [5]. In our patient, fMRI was not performed, and no medications were needed for treating his AHS because of rapid symptom improvement.

## Conclusions

Alien hand syndrome remains a conundrum of a symptom, which requires more research to fully understand the pathophysiology and intricate neuronal interconnections that may potentially help to establish future diagnostic tools, and assist with treatment. It remains a difficult diagnosis to make, and to manage, given its very low prevalence. Lastly, it is essential to note that even though alien hand syndrome is much more common in the left hand, it can occasionally occur on the right side as well. Furthermore, there are several different classifications available for AHS which need to be simplified.
